# Stay Put or Move Out? A Review of Acute Respiratory Illness Transfers Out of a Peripheral Centre and Their Characteristics: A Retrospective Cohort Study

**DOI:** 10.1111/jpc.70033

**Published:** 2025-03-13

**Authors:** James Pho, Alison Tran, Mani Suleiman, David Tran, Wei Qi Fan, Rebekah Barker, Rami Subhi

**Affiliations:** ^1^ General Paediatrics, Northern Health Melbourne Victoria Australia; ^2^ La Trobe University – Bundoora Campus Melbourne Victoria Australia; ^3^ The University of Melbourne – Parkville Campus Melbourne Victoria Australia; ^4^ Murdoch Children's Research Institute Melbourne Victoria Australia

**Keywords:** emergency medicine, general paediatrics, intensive care

## Abstract

**Aim:**

To describe the characteristics of children transferred out of a peripheral centre for the management of acute respiratory illness and explore opportunities for decentralising paediatric high dependency care.

**Methods:**

A single‐centre, retrospective cohort study, including children transferred out of a peripheral centre with diagnoses of asthma, bronchiolitis, pneumonia and pleural effusion. Patient characteristics and management were recorded from the medical records. Transfers were classified as ‘within scope’ and ‘outside scope’ of a peripheral paediatric centre. A multivariate regression was used to identify predictors of within scope transfers.

**Results:**

Between September 2015 and September 2023, there were 852 transfers, of which 165 (19.4%) met the study inclusion criteria. Ninety‐three (56.4%) transfers were within scope. Pre‐transfer diagnoses of preschool asthma, bronchiolitis, use of high flow nasal prong therapy (HFNP) and transfer directly from the emergency department (rather than ward) were more common for within scope transfers. HFNP was used in 103 (62.4%) of patients, including 74 (79.6%) of within scope transfers. Within scope transfers were predicted by preschool asthma (aOR 17.1, 95% CI 2.1–142.2, *p* = 0.01) and HFNP therapy pre‐transfer (aOR 5.4, 95% CI 2.2–13.2, *p* < 0.0001).

**Conclusion:**

This study shows a cohort of patients that could benefit from a model of care involving decentralising paediatric high dependency care, particularly children with preschool asthma, and those transferred for closer monitoring on HFNP therapy. More work needs to be done to define the scope of practice, guidelines and training, staffing and resourcing and systems for safety netting.

**Summary**

- What is already known on this topic
  ○ Paediatric intensive care unit (PICU) admissions have risen over the past 20 years, with increasing complexity and length of stay leading to strain on intensive care resources.
  ○ Respiratory illnesses are the most common diagnoses for patients to be admitted to the PICU.
  ○ There may be opportunities to decentralise aspects of high dependency care for children.
- What this paper adds
  ○ More than half of transfers out of peripheral paediatric centres do not receive interventions that could not be offered in the referring facility, beyond more intensive monitoring by trained nursing and medical staff.
  ○ Children with preschool asthma, and those managed with HFNP therapy pre‐transfer represent groups of children with respiratory illnesses for whom transfer may be prevented by strengthening high dependency capacity in peripheral centres.
  ○ More work needs to be done to define the scope of practice, guidelines and training, staffing and resourcing and systems for safety netting.

## 1 Introduction

Paediatric admissions to intensive care units (ICU) have risen over the last two decades, with an increasing complexity of case mix and longer lengths of stay [1]. Concurrently, centralisation of intensive care services for children within tertiary paediatric ICUs (PICU), which now receive > 90% of paediatric ICU admissions in Australia [2], has placed mounting pressure on PICU beds. There have therefore been calls to identify subgroups of children who can safely be cared for closer to home, in general ICUs or equipped paediatric high dependency units (HDUs) [3, 4].

Respiratory illnesses—bronchiolitis, pneumonia and asthma—are the most common non‐elective diagnoses of children admitted to PICU or a general ICU [5]. ICU admissions for bronchiolitis have doubled over the last 10 years, partly in association with increasing use of high flow nasal cannula (HFNC) and non‐invasive ventilation [6], and a reduction in mechanical ventilation [7]. Similarly, in a cohort of 14 029 children with asthma in Australia, 243 (1.7%) were admitted to ICU, but only 20 (0.1%) received CPAP or BiPAP, and 4 (0.03%) were mechanically ventilated [8]. These data suggest that there are opportunities to care for some of these children safely outside of tertiary PICUs.

We sought to describe the cohort of children transferred out of a large peripheral centre in Victoria for further management of acute respiratory infections. The primary aim was to characterise the subgroup of transfers that could potentially be managed within the scope of peripheral and regional paediatric services. The secondary aim was to investigate predictors of these transfers.

## 2 Methods

### 2.1 Setting

The Northern Hospital (TNH) is a large peripheral centre in Melbourne, with 2000 paediatric emergency department presentations and 200–300 admissions a month. There is a dedicated paediatric emergency department and inpatient ward. Children requiring HDU/ICU level care are transferred to tertiary centres, which are approximately 30 min away via road transport. There is access to paediatric anaesthetics during working hours, but no dedicated paediatric intensivists or paediatric sub‐specialty inpatient services on site. High flow nasal prong (HFNP) therapy can be provided in ED and on the ward. CPAP, Bilevel Positive Airway Pressure (BiPAP) and mechanical ventilation (MV) can be initiated in preparation for transfer. The nurse:patient ratio is up to 1:1 in a resuscitation emergency cubicle, 1:4 in admission wards and 1:8 overnight. On‐site radiology, including chest ultrasonography is available.

### 2.2 Design

We conducted a retrospective cohort study of children at TNH (emergency department and/or paediatric ward) between September 2015 and September 2023 who required transfer to a tertiary hospital for the management of a presumptive clinical diagnoses of lower respiratory tract infection or reactive airways. Ethics approval was obtained from the Northern Health Office of Research and Ethics (ALR 46.2023) prior to commencing data collection. We identified children with TNH discharge diagnoses that included ICD‐10 codes for acute bronchiolitis, asthma (which included preschool asthma/wheeze), pleural effusion and pneumonia. We cross‐checked these data with centralised records kept by the state's Paediatric Infant Perinatal Emergency Retrieval service (PIPER). We excluded transfers out of the special care nursery. All paediatric patients who met the above inclusion criteria were included in this study to minimise selection bias.

### 2.3 Data Collection

Electronic medical records were reviewed, and de‐identified data were collected from discharge summaries on transfer and on discharge from the receiving hospital. Data were extracted onto a REDCap form (Research Electronic Data Capture) and included demographic information, medical history, provisional diagnosis, medical therapies including respiratory support and timing and reasons for transfer. Data extracted from receiving hospital discharge summaries included final diagnosis, medical and surgical therapies, respiratory support, ICU level therapies (including inotropes, filtration and extra‐corporeal membrane oxygenation [ECMO]) and outcome. There was no missing data for all the variables of interest in this study.

### 2.4 Definitions

We defined preschool asthma as bronchodilator responsive illness in children ≤ 5 years, and asthma in those > 5 years. Transfers defined as ‘within scope’ of a paediatric centre are those for whom subsequent care in the receiving centre included low flow nasal prong oxygen (LFNP) or HFNP, and no requirement for inotropes, filtration, chest drainage, video assisted thoracoscopic surgery (VATS) or extra corporeal membrane oxygenation (ECMO). For the purposes of this study, we did not include human resources or capacity (e.g., nurse to patient or staff ratios) in this definition, but recognise that this is clearly a critical factor influencing decision‐making to transfer unwell patients to tertiary centres.

### 2.5 Statistical Analysis

Data were exported to Stata 17 for analysis [9]. We defined two groups: transfers with subsequent care in the receiving centre that fall within the scope of a peripheral centre, and transfers which required tertiary level management and fall outside the scope of a peripheral centre. We summarised baseline characteristics for each group using proportions (%), with *χ* ^2^ test (dichotomous variables) and Wilcoxon rank‐sum test of medians (continuous variables) with a *p* < 0.05 to reject the null hypothesis of no difference. We mapped respiratory support in the referring and receiving centres using a Sankey diagram.

We investigated predictors of transfers where subsequent care remained within the scope of a peripheral centre using a multi‐variable logistic regression, with stepwise forward selection (using a p‐value of 0.2) of the following variates identified a priori: age, sex, medical history and risk factors for severe illness or deterioration, pre‐transfer diagnosis, medical therapies pre‐transfer, respiratory support modality and location pre‐transfer (ED vs. ward). A receiver operating characteristic (ROC) curve analysis was undertaken, and the area under the curve (AUC) was calculated to assess model performance.

## 3 Results

Between September 2015 and September 2023, there were 852 total transfers to one of two tertiary paediatric centres. A total of 165 transfers (19.4%) met inclusion criteria. For 93 (56.4%) transfers, the therapies provided in the receiving centre were within the scope of a peripheral centre. Characteristics of transfers within and outside the scope are compared in Table 1. A history of asthma, difficulty breathing and pre‐transfer diagnoses of preschool asthma, bronchiolitis, use of HFNP and transfer directly from ED (rather than ward) were more common for within‐scope transfers. A history of fever, poor feeding and lethargy as well as diagnoses of pneumonia, pleural effusion and sepsis were more common for outside scope transfers. Discharge diagnoses from the receiving centres were bronchiolitis (*n* = 47, 31.3%), pneumonia (*n* = 41, 24.8%), pleural effusion (*n* = 29, 17.6%), preschool asthma (*n* = 26, 15.8%), asthma (*n* = 12, 7.3%) and sepsis (*n* = 10, 6.1%). Median (interquartile range) time from triage to decision to transfer was 5.9 (2.9–13.7) h for within scope and 9.8 (2.8–45.1) h for outside scope transfers (*p* = 0.16).

**TABLE 1:** Baseline characteristics, pre‐transfer diagnoses and therapies for children whose subsequent management in the referring centre falls within and without the scope of a peripheral centre. *p*‐value for *χ* ^2^ test.

	Within scope (*N* = 93)	Outside scope (*N* = 72)	*p*
Sex (female)	33 (35.5)	28 (38.9)	0.65
Age	15.6 (8.6–38.9)	19.1 (3.8–64.2)	0.76^a^
Time triage to referral for transfer, median hours (interquartile range)	5.9 (2.9–13.7)	9.8 (2.8–45.1)	0.14^a^
Risk factors			
Immunisations	88 (94.6)	64 (88.9)	0.28
History of prematurity	15 (16.1)	14 (19.4)	0.55
History of asthma	21 (22.6)	7 (9.7)	0.03
Immunocompromise	0	0	—
Neurodevelopmental condition	4 (4.3)	5 (6.9)	0.46
Genetic syndrome	5 (5.4)	4 (5.6)	0.96
Cardiac condition	7 (7.5)	4 (5.6)	0.62
Presenting symptoms			
Cough	65 (69.9)	44 (61.1)	0.24
Fever	24 (25.8)	29 (40.3)	0.05
Difficulty breathing	81 (87.0)	54 (75.0)	0.05
Poor feeding	17 (18.3)	26 (36.1)	0.01
Vomiting	7 (7.5)	12 (16.7)	0.07
Diarrhoea	1 (1.1)	2 (2.8)	0.42
Lethargy	7 (7.5)	14 (19.4)	0.02
Pre‐transfer location			
Emergency department	65 (69.9)	36 (50.0)	0.01
Inpatient ward	28 (38.9)	36 (50.0)	
Pre‐transfer diagnosis			
Preschool asthma	21 (22.6)	2 (2.8)	< 0.001
Bronchiolitis	43 (46.2)	23 (31.9)	0.06
Asthma	17 (18.3)	11 (15.3)	0.61
Pneumonia	31 (33.3)	38 (52.8)	0.01
Large pleural effusion	5 (5.4)	24 (33.3)	< 0.001
Sepsis	2 (2.2)	11 (15.3)	0.002
Reason for transfer			
Sub‐specialty review	45 (48.4)	31 (43.1)	0.78
Requires more intensive monitoring only	28 (30.1)	10 (13.9)	0.036
May need CPAP	20 (21.5)	18 (25.0)	0.72
On CPAP/BiPAP/MV	0	13 (18.1)	—
Highest level of respiratory support (pre‐transfer)			
Nil	6 (6.5)	7 (9.7)	0.56
Low flow oxygen	13 (14.0)	13 (18.1)	0.67
High flow nasal prongs	74 (79.6)	29 (40.3)	0.008
Continuous positive airway pressure	—	9 (12.5)	—
Bilevel positive airway pressure	—	1 (1.4)	—
Mechanical ventilation	—	13 (18.1)	—
Max FiO_2_ on HFNP (pre‐transfer)	40.5 (30–59)	50 (32–55)	0.48
Admission ward post‐transfer			
PICU	66 (71.0)	53 (73.6)	0.90
General medicine	14 (15.1)	1 (1.4)	0.005
Emergency cubicle	0	1 (1.4)	0.44
Short stay unit	6 (6.5)	0	0.04
Other specialty ward	7 (7.5)	14 (19.4)	0.06
Medical therapies (pre‐ and post‐transfer)			
Salbutamol	62 (66.7)	23 (31.9)	< 0.001
Ipratropium bromide	36 (38.7)	15 (20.8)	0.01
Steroids (any route)	38 (40.8)	8 (11.1)	< 0.001
Magnesium sulphate	31 (33.3)	12 (16.7)	0.02
Aminophylline	27 (29.0)	13 (18.1)	0.10
Adrenaline (nebules)	6 (6.5)	2 (2.8)	0.28
Inotropes/vasopressors	—	9 (12.5)	—
Filtration	—	4 (5.6)	—
Surgical therapies (post‐transfer)			
Chest drain	—	25 (34.7)	—
VATS	—	16 (22.2)	
ECMO	—	0	—

^a^ *p*‐value for Wilcoxon rank‐sum test of medians.

Care provided in receiving centres was within the scope of a peripheral centre for 45/76 (48.4%) of those transferred for sub‐specialty review, 28/38 (73.7%) of those transferred for additional monitoring and 20/38 (52.6%) of those transferred for possible requirement of CPAP (Table 1).

**FIGURE 1:**
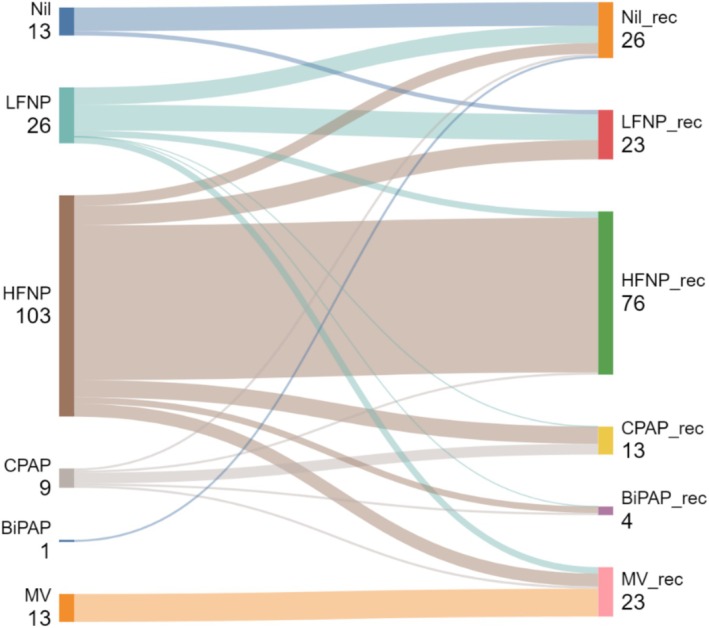
Sankey diagram showing highest respiratory support prior to and following transfer. BiPAP: bilevel positive airway pressure; CPAP: continuous positive airway pressure; MV: mechanical ventilation; VATS: video assisted thoracoscopic surgery.

In the receiving facilities, 119 (72.1%) were admitted to PICU, of whom 66 (55.4%) received within scope interventions. Figure 1 shows the highest level of respiratory support before and following transfer. HFNP (*n* = 103, 62.4%) was the most common form of respiratory support for transfer. At the receiving centres, HFNP was de‐escalated in 11 (nine LFNP, two no support) and escalated in 17 (eight CPAP, three BiPAP, six MV).

**FIGURE 2:**
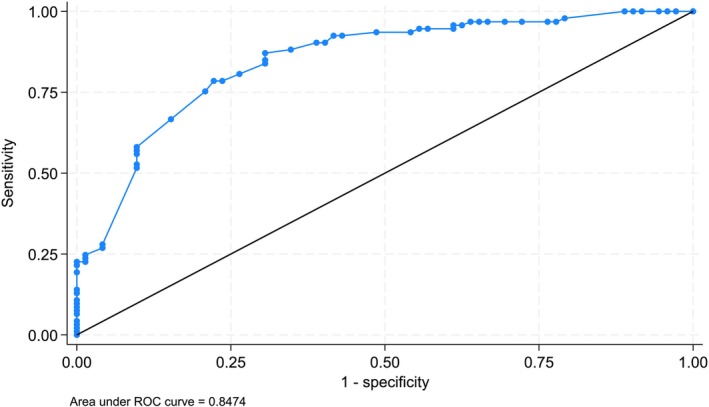
Area under the curve for multivariate regression.

Within scope transfers were predicted by a history of asthma (aOR 6.9, 95% CI 1.0–46.7, *p* = 0.05), a pre‐transfer diagnosis of preschool asthma (aOR 17.1, 95% CI 2.1–143.2, *p* = 0.01) and HFNP therapy pre‐transfer (aOR 5.4, 95% CI 2.2–13.2, *p* < 0.0001, Table 2). The AUC of the multi‐variable model was high (AUC = 0.847, Figure 2).

**TABLE 2:** Univariate and multivariable logistic regression of predictors of transfer where subsequent care in receiving centre was not outside scope of a peripheral centre.

	Univariate OR	*p*	Adjusted OR	*p*
Age	1.0 (0.99–1.0)	0.23	—	—
Male	1.2 (0.61–2.2)	0.65	—	—
Immunised	1.9 (0.60–6.4)	0.28	—	—
Preterm	0.78 (0.35–1.8)	0.55	—	—
History of asthma	2.7 (1.1–6.8)	0.03	6.9 (1.0–46.7)	0.05
Neurodevelopmental diagnosis	0.60 (0.16–2.3)	0.46	—	—
Genetic condition	0.97 (0.25–3.7)	0.96	—	—
Cardiac condition	1.4 (0.39–4.9)	0.62	—	—
Location pre‐transfer (reference ED)	0.43 (0.23–0.82)	0.01	0.56 (0.24–1.3)	0.17
Respiratory support pre‐transfer				
LFNP	1.2 (0.31–4.4)	0.82	—	—
HFNP	3.0 (0.9–9.6)	0.07	5.4 (2.2–13.2)	< 0.0001
Medical therapies				
MgSO_4_	2.5 (1.2–5.3)	0.02	6.9 (0.38–124.6)	0.19
Aminophylline	1.9 (0.88–3.9)	0.11	0.06 (0.002–1.4)	0.08
Nebulised adrenaline	2.4 (0.47–12.3)	0.29	—	—
Provisional (pre‐transfer) diagnoses				
Preschool asthma	10.2 (2.3–45.2)	0.002	17.1 (2.1–143.2)	0.01
Bronchiolitis	1.8 (0.96–3.5)	0.06	1.7 (0.43–6.6)	0.46
Asthma	1.2 (0.54–2.8)	0.61	—	—
Pneumonia	0.45 (0.24–0.84)	0.01	0.92 (0.31–2.7)	0.87
Pleural effusion	0.11 (0.04–0.32)	< 0.001	0.34 (0.08–1.4)	0.13
Sepsis	0.12 (0.03–0.57)	0.007	0.16 (0.02–1.1)	0.06

Abbreviations: aOR: adjusted odds ratio; HFNP: high flow nasal prong therapy; LFNP: low flow nasal prong oxygen; MgSO_4_: magnesium sulphate; OR: odds ratio.

## 4 Discussion

We conducted a retrospective cohort study of paediatric respiratory transfers out of the emergency department and inpatient ward of a large peripheral centre over 8 years (2015–2023). We found that transfers for acute respiratory infections account for 19.4% of all transfers, and for 56.4% of these transfers, care received following transfer remained within the scope of peripheral paediatric centres. In our cohort, although 71.5% of patients were admitted to PICU, 55.9% of these patients did not receive care that was outside the scope of a peripheral centre beyond intensive monitoring. We found that a pre‐transfer diagnosis of preschool asthma and HFNP therapy predicted transfers for whom care remained within the scope of a peripheral centre. A higher proportion of children with a pre‐transfer diagnosis of large pleural effusion and sepsis had outside scope transfers (pleural effusion: 33.3% vs. 5.4%; and sepsis 15.3% vs. 2.2%). These diagnoses predicted outside scope transfers in univariate regression, but the association was less pronounced in multivariate regression.

The decision to transfer children with acute respiratory illnesses receiving within scope interventions in peripheral EDs is usually triggered by abnormal observations on track and trigger charts [10], along with limited options for close observation outside ED resuscitation cubicles. These factors tend to result in an early decision for transfer (within a median of 5.9 h for transfers out of ED), without allowing an opportunity for stabilisation or response to initial treatment. In asthma, which accounted for 40% of within scope transfers in our cohort and predicted within scope transfers for preschool children, a period of up to 2 h is needed for intravenous bronchodilators [11], and 4 h for steroids to take effect [12]. This could contribute significantly to the 28.5% of our cohort who did not end up needing admission to PICU by the time they were received at their transfer site and suggests the need for a longer period of close observation for patients to declare themselves.

HFNP use pre‐transfer predicted within scope transfers (aOR 5.4, 95% CI 2.2–13.2). In our cohort, 62% of children were commenced on HFNP pre‐transfer, including 80% of within scope transfers. The majority (83%) of these children either remained on HFNP or had de‐escalation of respiratory support following transfer. Previous reports have shown that the rate of bronchiolitis admission to PICUs is rising without an increase in disease severity [13] in association with increasing utilisation of HFNP [14], and with opportunities to reduce PICU admission and hospital length of stay by reducing HFNP use [15]. For those on HFNP, the decision to transfer is influenced heavily by ED and ward‐based FiO_2_ cut‐offs (usually FiO_2_ > 0.4). Additional capacity to monitor children commenced on HFNP closely for longer periods, using not only an FiO_2_ at one point in time but also the pattern of clinical progression, including venous gas results, may help further risk stratify children who require transfer for respiratory support escalation.

The centralisation of paediatric intensive care and acute care services over the last 25 years followed from evidence showing higher odds of death in children cared for in non‐tertiary mixed adult/paediatric units, compared to centralised tertiary PICU [16]. But the epidemiology of PICU admissions has changed over time. With advances in neonatal and paediatric critical and surgical care, nutrition and gene therapy, more children are surviving with complex conditions [17], with an increasing proportion of ICU admissions with underlying comorbidities, baseline technology dependence (e.g., home NIV and feeding tubes) and longer hospital lengths of stay and costs [1]. Mounting pressure for PICU beds calls for a re‐examination of whether selected cohorts of otherwise well children with single disease processes can be safely cared for outside of a tertiary PICU. Models for this may include HDUs within tertiary centres, or co‐located within the paediatric wards or ICU units of peripheral centres. While some units in Victoria manage critically unwell children within general ICUs, there are no established models for HDUs outside of tertiary centres.

HDUs—a step down from PICU, a step up from general wards—can improve the quality of nursing care [18], patient and staff satisfaction [19], in addition to resource savings in PICU beds. A report of paediatric critical care services in the United Kingdom calls for distinguishing between critically ill children and those requiring enhanced nursing interventions, defining three levels of paediatric care: level 1 (all units admitting children), level 2 (more frequent monitoring and complex interventions), level 3 (PICU) [4]. In this study, we have identified opportunities to support the development of level 2 units within peripheral centres, but have distinguished between ‘within scope’ transfers and ‘avoidable transfers’, acknowledging that most peripheral paediatric units are not yet currently equipped. There remain gaps at peripheral centres in capacity for monitoring, technology (e.g., monitored beds), capacity and coverage of medical and nursing staff and relevant policies and procedures.

There are limited data describing acute care capabilities of peripheral paediatric centres in Australia, despite recent calls for centralisation of high dependency care [3]. Our study therefore fills an important gap in the literature. Limitations of this study include the retrospective design, reliance on documentation in medical records and small sample sizes of sub‐groups. We did not capture data on important determinants of the decision to instigate a transfer, particularly staffing and unit acuity, and our analysis cannot prove causal links. Future mixed methods research should probe peripheral centre preparedness and attitudes towards increasing the scope of practice for paediatric acute care. Data are from a single institution, reflecting the catchment's patient demographics, and the facility's organisational culture and local clinical practices, which will not be fully generalisable. This is particularly relevant for referral thresholds, which are influenced by many contextual factors, including referring unit capacity (space, staffing, systems), norms and rules, distances from tertiary centres and logistics of transfer.

## 5 Conclusion

This study shows a cohort of patients with acute respiratory illnesses that could potentially benefit from a model of care involving decentralising high dependency care, particularly children with preschool asthma, and those transferred for closer monitoring on HFNP therapy. More work needs to be done to define the scope of practice, guidelines and training, staffing and resourcing and systems for safety netting.

## Author Contributions

All authors have made substantial contributions to the work reported in this manuscript (e.g., technical assistance, writing or editing assistance, data collection, analysis).

## Conflicts of Interest

The authors declare no conflicts of interest.
